# Characterization of the chloroplast genome of the *Osmanthus didymopetalus*

**DOI:** 10.1080/23802359.2020.1778551

**Published:** 2020-06-19

**Authors:** Changheng Zhao, Qinghua Yang, Min Zhang, Cheng Zhang, Xiaofei Wang, Xianrong Wang, Yongfu Li

**Affiliations:** a College of Life and Environment Science, Huangshan University, Anhui, Huangshan, China; b Anhui Runyi Ecological Construction Co. Ltd, Anhui, Huangshan, China; c International Cultivar Registration Center for Osmanthus, College of Biology and the Environment, Nanjing Forestry University, Jiangsu, Nanjing, China; d Co-Innovation Center for Sustainable Forestry in Southern China, Nanjing Forestry University, Jiangsu, Nanjing, China; e Department of Bioengineering, Huangshan Vocational and Technical College, Anhui, Huangshan, China

**Keywords:** *Osmanthus didymopetalus*, plastid genome, genome structure, phylogeny

## Abstract

In this study, we assembled the chloroplast genome of *Osmanthus didymopetalus* (Oleaceae), a rare evergreen tree native to Hainan, China. The genome of *O. didymopetalus* was 155,155 bp in length and contained a pair of inverted repeats (IR, 25,697–25,704 bp) regions, which were separated by the small single copy (SSC, 17,591 bp) and the large single copy (LSC, 86,225 bp) regions. The cp genome encoded 133 genes including 88 protein-coding genes, 37 tRNA genes, and eight rRNA ribosomal genes. The overall GC content of *O. didymopetalus* chloroplast genome is 37.8%. Phylogenetic results showed that *O. didymopetalus* was more closely to *O. yunnanensis*, *O. fragrans* and *O. insulari*s. This study will be beneficial for the evolutionary study and phylogenetic reconstruction of *Osmanthus*.

The *Osmanthus* is a genus of flowering plant within Oleaceae. The species of this genus are considered to have high ornamental value in gardens owing to attractive elegant flowers with charming fragrance. *Osmanthus didymopetalus* P. S. Green, an evergreen tree with a height of 3–18 m, is restricted to Hainan province, China. Morphologically, it is the only species in this genus whose corolla lobes united in pairs at base, not forming a tube. At present, it has been classified as “Vulnerable, VU” in the Threatened Species List of China’s Higher Plants (Haining et al. 2017). While, there has been little research into the molecular biology or phylogenetic relationship of this species. Therefore, closely attentions should be paid on the genetic resources of this species to keep its sustainable development. For such a purpose, we assembled and characterized the complete chloroplast genome for *O*. *didymopetalus* from the genome skimming data in this study.

Fresh leaves of a single individual of *O. didymopetalus* were sampled from South China Botanical Garden, Chinese Academy of Science (23.188303°N, 113.378834°E). Total genomic DNA was isolated using the DNeasy Plant Mini Kit (Qiagen, Valencia, CA). The voucher specimen was deposited at the herbarium of Nanjing Forestry University (NF, accession number NF000096) and DNA compounds were stored at −20 °C, and was used to prepare the shotgun library following the manufacturer’s protocol for Hiseq4000 Sequencing System (Illumina, San Diego, CA). The library was sequenced by Nanjing Genepioneer. Biotechnologies Inc. (Nanjing, China). In all, 15,306,606 of PE150-bp raw reads were obtained, and were trimmed using CLC Genomics Workbench v9 (CLC Bio, Aarhus, Denmark) with default parameters. The resultant clean reads were then employed to assemble the chloroplast genome using the program NOVOPlasty (Dierckxsens et al. 2016) with *O. fragrans* (GenBank: MH687871) as the reference. A total of 116,174 individual reads yielded an average coverage of 185×. The resultant genome was annotated by CpGAVAS (Liu et al. 2012).

The circular genome of *O. didymopetalus* (GenBank: MT362090) was 155,155 bp in size, and contained two inverted repeat (IRa and IRb) regions of 25,697–25,704 bp, which were separated by a large single copy (LSC) region of 86,225 bp and a small single copy (SSC) region of 17,591 bp (Figure 1). A total of 133 genes are encoded, including 88 protein-coding genes (PCGs), 37 tRNAs and eight rRNAs. There was eight genes (*rpl2*, *rpl23*, *ycf2*, *ycf15*, *ndhB*, *rps7*, *rps12*, *ycf1*) located in the IRs region. The *rpl2* located in the boundary of LSC and IRb, while *ycf1* located in the border of SSC and IRa. Most of genes occurred in a single copy; however, four protein-coding genes (*ndhB*, *rpl2*, *rpl23*, *rps7*), eight tRNA genes (*trnA-UGC*, *trnG-GCC*, *trnI-CAU*, *trnI-GAU*, *trnL-CAA*, *trnN-GUU*, *trnR-ACG*, *trnV-GAC*) and four rRNA genes (*rrn4.5*, *rrn5*, *rrn16S*, *rrn23*) are duplicated. The overall GC content of *O. didymopetalus* genome is 37.8% and the corresponding values in LSC, SSC and IR regions are 35.8%, 32% and 43.2%, respectively. Four genes, *atpF*, *ndhA*, *rpoC1*, *rps16* have a single intron, while the other five genes, *clpP*, *ndhB*, *rpl2*, *rps12*, *ycf3* harbor two introns.

**Figure 1.**
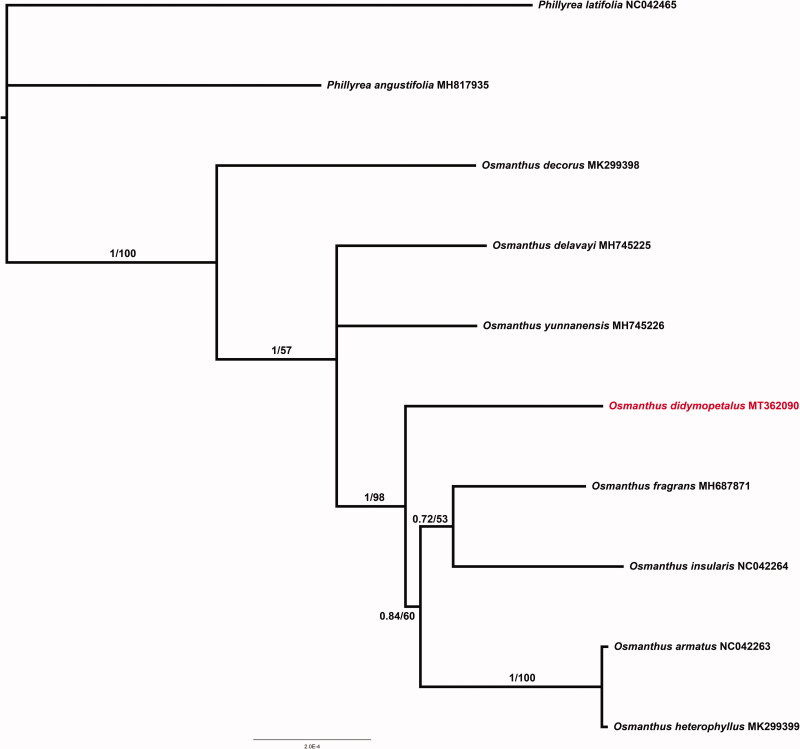
Phylogenetic tree inferred from10 complete cp genomes. The support value is displayed above the branch in the order of posterior probability from BI, likelihood bootstrap values from ML.

The phylogenetic analysis was conducted based on nine *Osmanthus* cp genomes and two taxa within *Phyillyrea* (*P. angustifolia* and *P. latifolia*) as outgroups. All of the chloroplast genome sequences were aligned using MAFFT (Katoh and Standley 2013). The maximum likelihood (ML) phylogeny was reconstructed using IQ-tree (Nguyen et al. 2015) with 10,000 bootstraps (BS) under the TVM + R2 + F substitution model. Bayesian inference (BI) analyses were conducted using Mrbayes v 3.2.6 (Ronquist and Huelsenbeck 2003) under GTR + I + G substitution model with two independent Markov chain Monte Carlo (MCMC) analyses run, each with three heated and one cold chain for 10,000 generations. The phylogenetic trees revealed that accessions from *Osmanthus* formed a monophyletic clade, and *O. didymopetalus* was suggested more closely to *O. yunnanensis*, *O. fragrans* and *O. insularis* (Figure 1).

## Data Availability

The data that support the findings of this study are openly available in NCBI at https://www.ncbi.nlm.nih.gov/.
